# Emerging role of UFMylation in secretory cells involved in the endocrine system by maintaining ER proteostasis

**DOI:** 10.3389/fendo.2022.1085408

**Published:** 2023-01-19

**Authors:** Yun Cheng, Zikang Niu, Yafei Cai, Wei Zhang

**Affiliations:** ^1^ Jiangsu Cancer Hospital, Jiangsu Institute of Cancer Research, The Affiliated Cancer Hospital of Nanjing Medical University, Nanjing, China; ^2^ College of Animal Science and Technology, Nanjing Agricultural University, Nanjing, China

**Keywords:** UFMylation, secretory pathway, endocrine, ER proteostasis, pancreas, ovaries

## Abstract

Ubiquitin-fold modifier 1 (UFM1) is a ubiquitin-like molecule (UBL) discovered almost two decades ago, but our knowledge about the cellular and molecular mechanisms of this novel protein post-translational modification is still very fragmentary. In this review, we first summarize the core enzymes and factors involved in the UFMylation cascade, which, similar to ubiquitin, is consecutively catalyzed by UFM1-activating enzyme 5 (UBA5), UFM1-conjugating enzyme 1 (UFC1) and UFM1-specific ligase 1 (UFL1). Inspired by the substantial implications of UFM1 machinery in the secretory pathway, we next concentrate on the puzzling role of UFMylation in maintaining ER protein homeostasis, intending to illustrate the underlying mechanisms and future perspectives. At last, given a robust ER network is a hallmark of healthy endocrine secretory cells, we emphasize the function of UFM1 modification in physiology and pathology in the context of endocrine glands pancreas and female ovaries, aiming to provide precise insight into other internal glands of the endocrine system.

## 1 Introduction

Ubiquitin was first identified in the 1970s as a small ubiquitous protein (8.5 kDa) that is evolutionary conserved in eukaryotes (1, 2). Ubiquitylation (or ubiquitination) was subsequently shown to be brought about by E1 (activating enzymes), E2 (conjugating enzymes), and E3 (ligase enzymes) (3). These enzymes catalyze the formation of a peptide bond between the C-terminal glycine of ubiquitin and typically a lysine residue on a substrate protein (4). Besides ubiquitin, a large family of ubiquitin-like proteins (Ubls) has been discovered in the 1990s and 2000s (5). Although they do not necessarily possess a high degree of sequence similarity to ubiquitin, these Ubls all share the ubiquitin fold and the ability to be conjugated to substrates through the concerted action of evolutionarily related E1s, E2s, and E3s (6). Altogether, ubiquitin and ubiquitin-like molecules constitute the third most common type of post-translational modification after phosphorylation and glycosylation (7).

**Figure 1:**
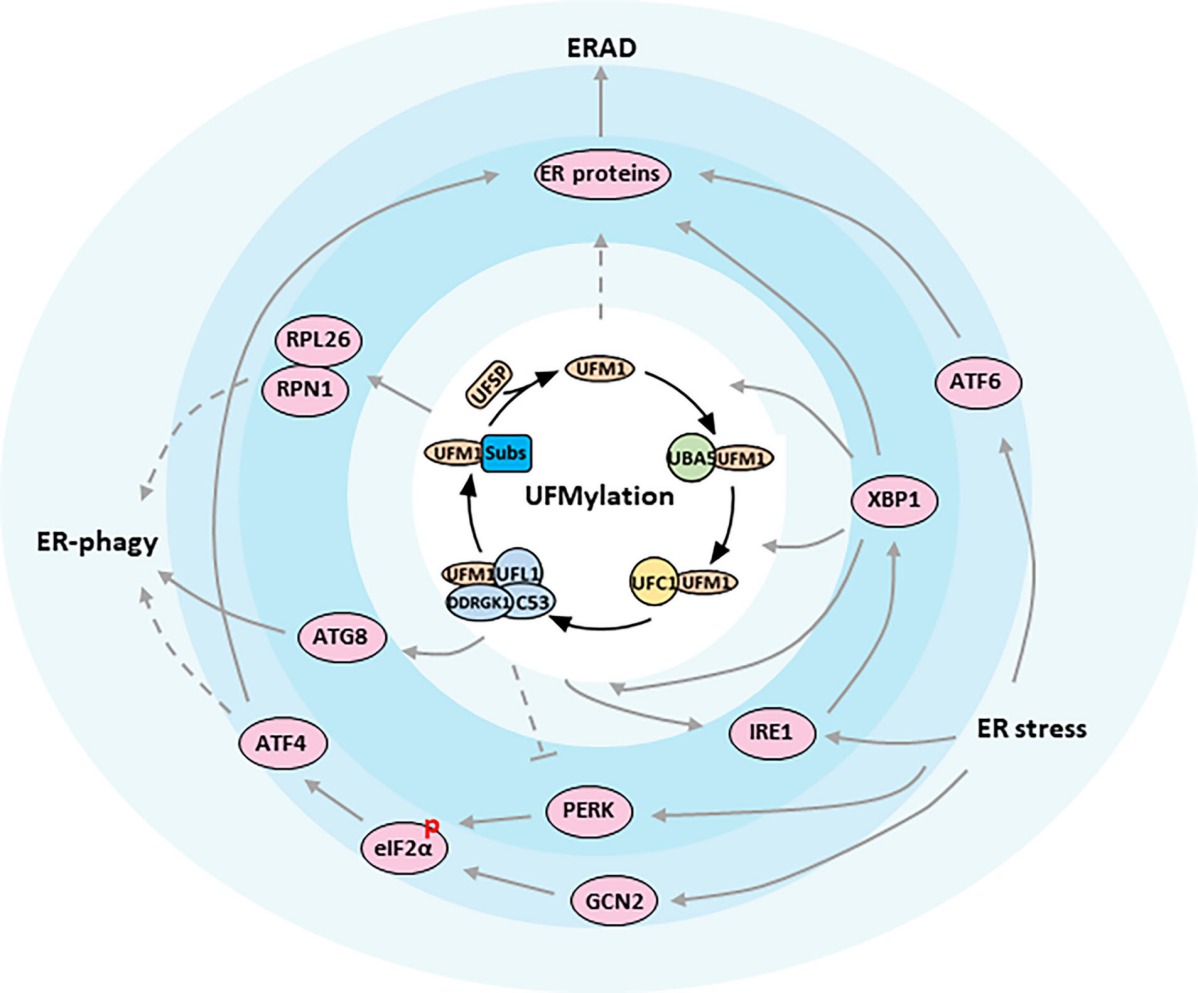
ER proteostasis mechanisms of UFMylation. Functional relationships between components are indicated. The figure presents key components of the UFMylation pathway in the center of the regulatory network. Arrows indicate activation whereas bar-ended lines indicate inhibitory interactions. Broken lines indicate indirect interactions or interactions requiring further study.

While the ubiquitin conjugation cascade has been extensively studied over the past few decades, the functions of many other Ubls still remain unclear. One such modifier is the ubiquitin-fold modifier 1 (UFM1), which was originally found in 2004, and is evolutionarily expressed in metazoa and plants except in fungi (8, 9). Produced as a precursor containing 85 amino acids (9.1 kDa), UFM1 is first activated by two specific cysteine proteases, UFSP1 and UFSP2, to expose the conserved glycine required for covalent attachment to target proteins through removing the extra two amino acids at the C-terminal region (resulting in 83 amino acids, 8.9 kDa) (10, 11). After maturation, UFM1 then elicits conjugating reactions involving a three-step enzymatic cascade successively catalyzed by UFM1-activating enzyme 5 (UBA5, E1), UFM1-conjugating enzyme 1 (UFC1, E2), and UFM1-specific ligase 1 (UFL1, E3) (12–14). Similar to Ubiquitylation, the UFM1 modification process outlined above is often referred to as UFMylation, which interestingly is reversible because of the UFSPs mediated de-UFMylation to separate UFM1 from its target (11) (**Figure 1**).

The physiological importance of UFMylation has been evidenced by dysregulations of the pathway components that lead to diseases such as cancer, heart failure, gut inflammation and neurodevelopmental disorders (15–18). Yet the broad biological functions and working mechanism of the UFM1 system are still largely uncertain. Recent studies linking UFM1 to the ER and the secretory pathway highlight a potential mechanism to explain the essential role of UFMylation in tissue homeostasis and cell development (19).

The endocrine system is a messenger system comprising communications between organs and tissues *via* hormones released by internal glands, such as pancreas and ovaries, into the circulatory system (20). Considering a highly-developed ER is responsible for hormones production and secretion in endocrine glands, the goal of this manuscript is to provide an overview of key regulatory aspects of UFMylation, and focus on its close connection with ER homeostasis, especially in pancreas and ovaries. We hope this review will be helpful to inspire new ideas and new discoveries of UFM1 in other endocrine organs including the pituitary gland, adrenal glands, testis and so on.

### 1.1 UBA5 and UFC1

Although Ubiquitylation encompasses two E1s and at least ten E2s, there is solely one of each enzyme reported to date for the downstream UFMylation cascade (21). Following UFM1 maturation, UBA5, which acts as the E1 and initiates the conjugation reaction, forms a thioester bond with UFM1 *via* adenylation in an ATP-dependent manner. In the second step, UFM1 is transferred from UBA5 to UFC1, the E2, using a thioester linkage as well (22).

While most components of the UFM1 pathway, as discussed later, are mainly localized at the cytosolic side of the ER membrane, both UBA5 and UFC1 exhibit a cytosolic and also nuclear distribution (9, 21). Earlier studies in fibroblasts revealed that UBA5 mutations impair the process of UFMylation, resulting in an abnormal ER structure. Experimental and clinical findings from animal models and humans further support UBA5 aberrance as a pathophysiological cause for many abnormalities during neurodevelopment (23, 24). In addition to UFM1, UBA5 was also reported to conjugate SUMO2, another ubiquitin-like protein, and activate SUMO2 in the nucleus, though details of this interaction remain to be elucidated (21). Meantime, biallelic UFC1 variants disrupting UFM1-UFC1 formation have been identified in children with severe early-onset encephalopathy, also arguing for an essential role for UFMylation in brain development (18). Moreover, cell adhesion molecules NCAM140 and KIRREL3 were found to interact with UFC1, implicating a potential function of UFM1 modification for cell surface proteins (25, 26).

### 1.2 UFL1 and its cofactors

In contrast to the more than 600 ligases utilized by Ubiquitylation reaction, the ligation of UFM1 to its target proteins is mediated by UFL1, again, the only E3 defined thus far for UFMylation (14). Surprisingly, UFL1 does not have any typical domains conserved in other E3s except for a highly conserved N-terminal region across species. For this reason, it was believed that UFL1 may be a scaffold-type E3 that brings UFM1 substrate and UFC1 in close proximity, and the E3 ligase activity of UFL1 requires the assistance of other proteins (27). Indeed, evidence is growing that UFL1 forms a tripartite complex with DDRGK1 and C53 proteins, which are regarded as substrate adapters and key regulators for UFMylation system (28, 29) (**Figure 1**).

Intriguingly, interaction with UFM1 not only stabilizes DDRGK1 and C53, but also provokes their post-translational modifications by UFMylation. In fact, DDRGK1 (also known as C20orf116 and UFBP1) was the first characterized substrate of UFM1, and soon emerged as an E3 cofactor, whose UFMylation is not necessary for E3 complex assembly, but could alter the UFMylation of other substrates, such as ASC1, a nuclear receptor co-activator vital for breast cancer development (14, 28) (**Table 1**). While DDRGK1 possesses a proteasome-COP9-initiation factor (PCI) domain that embraces the major lysine site for UFMylation and mediates the binding with UFL1, both DDRGK1 and UFL1 contain a transmembrane peptide and a nuclear localization signal (NLS) with the latter likely working merely when the transmembrane domain is deleted (34, 35). Due to the transmembrane helix at its N-terminus, DDRGK1 is predominantly tethered to the ER membrane and recruits UFL1 and C53 to form a heteromeric complex at the cytosolic surface of ER (30, 36, 37). Defect in this hydrophobic N-terminal region of DDRGK1 would lead UFL1 to localize in the cytoplasm, where UFL1 was found to be cleaved with unknown mechanism (30). In addition, numerous functions have been described for DDRGK1, including ER homeostasis, stress responses and cell differentiation (38–40). However, it is still undetermined whether all these functions involve UFMylation.

**Table 1:** Well-characterized UFMylation substrates to date. .

UFMylated protein	Biological functions	Subcellular locations	Original studies
ASC1	originally identified as thyroid hormone receptor interactor 4 (TRIP4); a transcriptional coactivator of Estrogen receptor-α (ERα) for breast cancer development	nucleus and cytosol	(28)
C53	first identified as a binding protein of CDK5 activator; regulating cell cycle, cell survival, cell adherence/invasion, tumorigenesis and metastasis	cytosol, nucleus and ER	(27, 30)
DDRGK1	substrate adapter for UFMylation; plays a role in cartilage development through SOX9, which may be unrelated to UFMylation	ER	(14)
Histone H4	histone protein UFMylated for amplification of ATM activation following DNA damage	nucleus	(31)
MRE11	component of the MRN complex, which plays a central role in DSB repair *via* activation of ATM	nucleus	(32)
P53	containing transcriptional activation, thereby inducing cell cycle arrest, apoptosis, senescence, DNA repair or changes in metabolism	cytosol, nucleus, mitochondrion and ER	(15)
RPL26	component of the large ribosomal subunit	cytosol and ER	(29)
RPN1	ER quality-control factor that is part of the oligosaccharyltransferase complex	ER	(33)

The second putative adaptor protein for UFL1 is C53 (also named CDK5RAP3 or LZAP). Initially classified as a tumor suppressor, C53 was observed to translocate from ER to nucleus upon UFL1 depletion to inhibit cyclin D1 and cell cycle progression in glioma cells (41). This phenomenon probably was resulted from the direct binding of C53 to RelA. RelA is the regulatory subunit of NF-κB in nucleus, essential for the transcriptional activity of NF-κB, which is crucial for cyclin D1 expression, tumor formation and metastasis (42). Conversely, knockdown of UFL1 has also been documented to elicit elevated NF-κB activity and cell invasion in Hela and U2OS cells. Surprisingly the efficacy of UFL1 ablation on NF-κB was stronger than C53 knockdown, indicating, besides C53, UFL1 may also modulate other components of the NF-κB pathway (30). More controversially, an article by Xi and coworkers depicted that DDRGK1 could positively regulate the degradation of IκBα, the inhibitor of NF-κB, and consequently facilitate NF-κB activity (40). In short, the disagreement in this scenario was suggestive that components of the UFM1 pathway may have function independent of UFMylation, and the precise relationship among UFL1, DDRGK1 and C53 is still a mystery.

## 2 UFMylation maintains ER proteostasis

In vertebrates, endocrine glands are specialized organs that secrete hormones, either nitrogenous hormones or steroid hormones, into the endocrine signaling system. At the cellular level, the ER is the principal organelle that is responsible for the synthesis, folding, post-translational modifications, and secretion of proteins, as well as biosynthesis of lipids and sterols. It is estimated that the ER faces a heavy task to handle up to one million client proteins per minute per cell. The process, unfortunately, is error prone and any possible mistake is potentially adverse to the cells. Therefore, the ER is constantly monitored by multiple quality control mechanisms, consisting of autophagic degradation of the ER (termed as ER-phagy), ER-associated degradation (ERAD), and the unfolded protein response (UPR) (43) (**Figure 1**).

### 2.1 UFMylation regulates ER-phagy

In steady state, the ER undergoes continuous renovation to sustain proteostasis, which is achieved by two major mechanisms, ER-phagy and ERAD, to eliminate defective polypeptides. It is well-known that arrested protein products in the cytosol rendered by ribosome stalling are degraded by the ubiquitin-proteasome system. On the other side, removal of translation-faulty ER proteins requires lysosome (or vacuole in plants) associated ER-phagy, in that arrested proteins during co-translational protein translocation are situated inside the translocon with their N-termini inside the ER lumen (44, 45). Thus ER-phagy is usually defined as the lysosomal degradation of part of the ER network containing membranous compartments *via* partially or no intersecting with the selective autophagy machinery.

Recently a genome wide CRISPR screen identified UFL1 and DDRGK1, but not C53, as critical regulators for starvation-induced ER-phagy (33). They unveiled that nascent proteins trapped in the ribosome stimulates UFMylation of ER substrates such as RPN1 and RPL26 (**Table 1**), thereby targeting ER sheets for degradation through ER-phagy, though the downstream played by UFMylated RPN1 and RPL26 during ER marking and autophagic engulfment remain to be investigated (33) (**Figure 1**). On the contrary, C53 in plants and in human cell lines was justified to mediate ER stress-induced autophagic flux upon ribosome stalling after C53 was recruited to the ER by forming a functional ternary complex with UFL1 and DDRGK1. A working model was proposed simultaneously that UFM1 was transferred from C53 to RPL26 under proteotoxic stress so that C53 could act as an autophagy receptor to interact with ATG8 (Autophagy-related gene 8), another ubiquitin-like protein, and initiate autophagosome formation (46) (**Figure 1**). By comparing the two studies, the involvement of C53 in ER-phagy seems dependent on the physiological context, since phenotyping experiments revealed that C53 mutant was asymptomatic during carbon or nitrogen starvation but was highly sensitive to ER-stress treatments (46, 47). In that case, an in-depth exploration then is urgently needed to address whether UFMylation of DDRGK1 and C53 is a collateral effect or is in turn required for the further UFMylation of other substrates.

### 2.2 UFMylation and ERAD

Misfolded proteins in the secretory pathway ordinarily are monitored by molecular chaperons and eliminated *via* the ERAD system to ensure that only correctly folded proteins exit the ER (48, 49). When a protein fails to acquire its correct conformation, it is switched to the ERAD pathway and is immediately transported over the ER membrane towards the cytosol, where the ERAD substrate is ubiquitinated and degraded by the proteasome. Although significant progress has been made in dissecting the role of ERAD for ER homeostasis, little is known about the concrete link between UFM1 modification and ERAD. A study by Schuren, et al. aiming to isolate novel factors associated with ERAD reported that nearly all components in UFMylation affected the degradation of ER-resident HLA-I, a protein for cell immunity, in a different manner than ubiquitin (50). They uncovered that interference with the UFM1 conjugation machinery specifically inhibited the ER-to-cytosol dislocation of HLA-I and misdirected the ERAD-mediated protein sorting. Notwithstanding, they failed to detect any UFMylated substrate directly contributing to ERAD, in accordance with the ERAD phenotype in K562 and U2OS cells, which has been hinted as an indirect consequence of aberrant ER protein biogenesis impacted by erroneous UFMylation activities (33) (**Figure 1**). On the other hand, it will be intriguing to examine whether the same side effect also occurs to hormone production and secretion in endocrine glands.

### 2.3 UFMylation is intricately connected to ER stress

Various pathological conditions, such as nutrient deprivation, hypoxia, lipotoxicity, calcium depletion and viral infection, could perturb ER homeostasis (51–53). When the regulatory capacity of ER-phagy and ERAD is exhausted, misfolded proteins and their aggregates accumulate and trigger ER stress (49). To cope with this circumstance, cell has evolved a set of adaptive reactions, collectively called the unfolded protein response (UPR) that halts protein translation, enhances ER-folding capacity and elevates the activity of ER-phagy and ERAD. While temperate ER stress has the ability to protect cell and restore homeostasis, excessive or prolonged UPR would cause cell apoptosis and tissue damage (54).

There are three parallel branches of the UPR that are initiated by distinct ER stress sensors, including the kinase/RNase IRE1 (a synonym for ERN1, Endoplasmic reticulum to nucleus signaling 1), eIF2α kinases PERK and GCN2, and transcription factor ATF6 (55–59) (**Figure 1**). IRE1 is the most conserved sensor coordinating the UPR from the early stage of stress response. IRE1 oligomerizes upon ER stress and allows for trans-autophosphorylation and activation of its endonuclease domain, which splices the mRNA of the transcription factor XBP1 to drive the expressions of genes encoding chaperones, foldases, ERAD components and lipid synthesis enzymes (60–65). When restoration of ER homeostasis fails, IRE1 also represses the adaptive cellular responses and activates apoptosis through JNK signaling (66–68). The second branch of the UPR is dominated by PERK, which phosphorylates eIF2α and attenuates the initiation of global protein translation (53). Paradoxically, phosphorylation of eIF2α could preferentially translate the transcription factor ATF4, which subsequently regulates several target genes, such as CHOP involved in cell death (69). Distinct from PERK that shares similar ER domains to IRE1, cytoplasmic GCN2 has also been showed to phosphorylate eIF2α cooperatively with PERK, but it is still questionable how it communicates stress signals from the ER (58). Additionally, PERK is known to regulate proliferation and differentiation of insulin-secreting β-cells of endocrine pancreas, as well as to promote adipocyte differentiation *via* intrinsic lipid kinase activity. Both, however, might be unrelated to the UPR pathway. The last transducer is ATF6 that is released from ER under stress boost. ATF6 controls gene expressions in nucleus to increase ER volume and reinforce the processing and degradative capacities of ER, serving overlapping functions with IRE1 and PERK (70).

So far, there has been a persuasive accumulation of data implying that UFMylation has an intricate relation to ER stress and UPR. For example, Zhang, et al. showed that UFMylation genes were transcriptionally up-regulated by disturbance of the ER homeostasis and inhibition of vesicle trafficking. They illustrated UFM1 promoter contained a putative binding site for the ER stress-responsive transcriptional activator XBP1 (71). UFM1 system thereafter was indicated to be indispensable for ER stress tolerance, exerting a protective role to prevent cells from death in the presence of stress (72). Yet in sharp contrast, skin fibroblasts from individuals with mutations in UBA5 were found to be aberrant in UFMylation and resistant to ER stress-induced toxicity with abnormally expanded ER structure (73). These conflicts in general may be because UFM1 modification differentially influences the ER network in different cell types, as in a subset of cultured cells, targeted disruption of genes encoding UFM1 machinery only weakly activates an ER stress response but would prompt UPR and ER stress-induced apoptosis in cells like U2OS, hematopoietic stem cells (HSC) and pancreatic beta cells.

Mechanistically, Liu, et al. claimed that DDRGK1 could regulate IRE1 protein stability through an UFMylation-dependent protein-protein interaction (74) (**Figure 1**). They reported that depletion of DDRGK1 diminished IRE1-XBP1 signaling and activated the PERK-eIF2α-CHOP apoptotic pathway by targeting IRE1, probably owing to the similar induction requirements for the two UPR branches. As a result, absence of DDRGK1 in HSCs and cancer cells would lead to ER stress and finally disturb ER homeostasis. Nevertheless, neither the DDRGK1-mediated UFMylation nor stabilization of IRE1 can be verified by other scholars applying different cell lines and experimental settings (33, 75). Instead, higher expression and activation of IRE1-XBP1 was observed by Zhu et al. when DDRGK1 was deficient in plasma cell, an antibody secreting cell derived from B cell (75). Consistent with earlier literatures (36, 71), Zhu and his colleagues confirmed that the IRE1/XBP1 axis also upregulated the expression of DDRGK1 and UFM1 pathway genes in the differentiated B cell. They proved that DDRGK1 downstream of XBP1 was fundamental to the secretory function of plasma cell, and that lack of UFMylation retarded immunoglobulin production and impaired ER expansion, which likely fed back to the expression and activation of IRE1. In line with Liu’s speculation, however, they also suggested that DDRGK1 was required for the suppression of PERK during plasma cell development through an unknown mechanism but independent of DDRGK1 UFMylation.

Besides IRE1 and PERK, until now it is still not sure whether the UFM1 process interacts with GCN2 or ATF6 as well. Taken together, there definitely is a long way before the complicated interconnection between UFMylation and ER stress can be fully understood.

## 3 UFMylation in endocrine glands

As previously alluded to, it is predictable that UFMylation is intimately linked to the secretory pathway of endocrine secreting cells, in which optimal functioning of the ER is critical for proper proteostasis and cell survival. Indeed, components of the UFM1 conjugation pathway are remarkably abundant in pancreatic islets, male testis and some other endocrine tissues (36). Accordingly, although the role of UFMylation in endocrine cells has not been deeply surveyed (see **Table 2**), we are convinced that this is an emerging area that will offer a fruitful source for new ideas and discoveries in the near future. The following sections detail our current knowledge on pancreas and ovaries, two systems in which UFMylation was demonstrated to play a prominent role.

**Table 2:** Summary for UFMyation components in endocrine glands.

Endocrine glands	Literature	Research models	Phenotypes or mechanisms	Publication data
adrenal gland	(36)	mouse	Expression of UFM1 mRNA and protein in adrenal gland	2011
			Expression of DDRGK1 mRNA in adrenal gland	
			Expression of UFL1 mRNA in adrenal gland	
			Expression of C53 mRNA in adrenal gland	
hypothalamus/pituitary	(76)	chicken	Expression of UFM1 mRNA in hypothalamus and pituitary gland	2006
	(77)	chicken	UFM1 transcript in hypothalamus and pituitary was related to high egg production	2007
ovaries	(78)	bovine	Expression of UFL1 protein in ovaries	2020
			UFL1 regulates LPS-induced apoptosis in bGCs by inhibiting NF-κB Pathway	
	(39)	goat	Expression of UFM1 and DDRGK1 proteins in ovaries	2021
			Viability of GCs was not affected after overexpression of UFM1	
			UFM1 silencing inhibited GC proliferation and enhanced ER stress-induced apoptosis	
	(79)	mouse	UFL1 relieves ER stress and apoptosis in GCs, and protects ovary from cisplatin-induced damage	2022
pancreas	(80)	human	Expression of C53 mRNA in pancreas	2000
	(81)	MKR mouse	UFM1 for the first time was identified to be associated with islet dysfunction	2008
	(36)	mouse and INS1-832/13 cell line	Expression of UFM1 mRNA and protein in pancreas Expression of DDRGK1 mRNA in pancreas Expression of UFL1 mRNA in pancreas	2011
			Expression of C53 mRNA in pancreas	
			UFM1 and DDRGK1 are not involved in glucose stimulated insulin secretion	
			ER stress-induced apoptosis is increased after UFM1, DDRGK1 and UFL1 knockdown	
	(82)	db/db mouse	Lv-UFM1 (lentiviral overexpression of UFM1) exhibited more disrupted islet	2020
			integrity with uneven boundaries	
			Lv-shUFM1 (UFM1 knockdown by lentivirus shRNA) showed significant improvement in pancreatic pathology	
			Knockdown of UFM1 also led to a reduced number of infiltrating macrophages in the pancreas	
testis	(36)	mouse	Expression of UFM1 mRNA and protein in testis	2011
			Expression of DDRGK1 mRNA in testis	
			Expression of UFL1 mRNA in testis	
			Expression of C53 mRNA in testis	
thymus	(36)	mouse	Expression of UFM1 mRNA and protein in thymus	2011
			Expression of DDRGK1 mRNA in thymus	
			Expression of UFL1 mRNA in thymus	
			Expression of C53 mRNA in thymus	
thyroid	(83)	human	A patient was suggestive of C53 positive anaplastic thyroid carcinoma, which had migrated to stomach	2019
	(84)	PTC cell line	C53 is a putative tumor suppressor in papillary thyroid carcinoma (PTC)	2022

### 3.1 UFMylation in pancreatic beta cells

The pancreas is well-known for its dual function - the exocrine function engaged in digestive enzymes active in the small intestine, which are pivotal for nutrient digestion, and the endocrine effect, which is stimulated to control over the blood glucose level *via* pancreatic hormones (85). Actually UFM1 system appears to be involved in both exocrine and endocrine cells. In light of the theme of this review, merely endocrinal regulation in pancreatic beta cell will be discussed below.

Compared with other endocrine hormones that are ultimately guided by the pituitary gland, the pancreatic beta cell is unique in its capacity to synthesize, store and secrete insulin with precise rates in response to circulating sugar, covering the metabolic needs of the organism (86). The fine-tuning of insulin production and secretion is modulated at multiple levels, ranging from transcription, mRNA stability to translation and protein folding. To fulfill the task of insulin biosynthesis, the beta cell has evolved a rich ER whose pathologies are tightly associated with metabolic disturbance and obesity. PERK null mice, for instance, have increased beta cell apoptosis and early onset diabetes, whereas CHOP deletion mice have improved beta cell function and cell survival when insulin demand rises (87, 88). Reciprocally, ER stress is activated under circumstances of cellular nutrient overload, including lipotoxicity (89). Genetic or diet-induced models of obesity would act *via* PERK to enhance obesity-induced insulin resistance, in which IRE1 was also shown to lower insulin responsiveness (90).

Notably, in diabetic MKR mice expressing dominant-negative mutant IGF-IRs specifically in skeletal muscle, Lu et al. observed marked up-regulation of UFM1 and ERAD proteins for the first time in pancreatic islets using a quantitative proteomics approach combined with microarray. They hence identified UFM1 as a potential factor for the development of type 2 diabetes (81). Thereafter, Lemaire et al. testified that UFM1 and DDRGK1 were mainly expressed in beta cells in the mouse islets of Langerhans. Their expression in pancreas was increased upon feeding and ER stress but not on other stressors, such as cyclohexamide and H_2_O_2_ (36). Furthermore, evidence was provided that failing UFMylation by RNAi triggers ER stress-induced apoptosis of beta cells although neither UFM1 nor DDRGK1 was required for the glucose-mediated insulin release.

### 3.2 UFMylation in ovaries

The ovary is the female gonad whose major function is the differentiation and release of mature oocytes for fertilization. It’s in charge of the synthesis and secretion of steroid hormones, including estrogen and progesterone, allowing the development of female secondary sexual characteristics and supporting pregnancy. In mammals, each follicle, the functional unit of ovary, is composed of an innermost germ cell (so called oocyte), surrounding granulosa cells (GCs), and outer layers of thecal cells (91). Controlled by endocrine and paracrine factors, the follicles develop with the proliferation of GCs through primary, secondary and antral phases, during which most follicles undergo atretic degeneration with a few of them eventually reaching the point of ovulation (39). After the secondary follicular stage, more protein synthesis and post-translational modifications are demanded in order to satisfy the accelerated GC proliferation and oocyte growth, which may create a deleterious situation for the ER (92–94). The follicular atresia thereby is initiated by GC apoptosis commonly thought to be evoked by the UPR pathway. By comparing the expression of UFL1 in mouse ovaries at different developmental stages, it was speculated that the increased expression of UFL1 during follicular development must dedicate to the maintenance of ER homeostasis in GCs, providing a stable internal environment for the maturation of follicles (95). Consistently, the expression levels of UFM1 and DDRGK1 were reported to be higher in GCs derived from antral atretic follicles than healthy ones in ovaries from ruminants (39).

In human, ovarian disease has gradually turned into hot topic due to the tendency of delaying the age of having children. Premature ovarian failure (POF) is a gynecological syndrome defined as an ovarian functional defect occurring before the age of 40 years. It is characterized by amenorrhea, hypogonadism and estrogen deficiency, severely reducing the fertility of women, especially in female cancer patients receiving chemotherapy drugs (96). A mouse model of POF was set up by intraperitoneal injection of cisplatin. In this model, cell apoptosis and follicular atresia were triggered by elevated ER stress in GCs associated with a significant decrease of UFL1 protein expression there (79). Interestingly, overexpression of UFL1 could alleviate ER stress and reduce cisplatin-induced damage to the ovary, in the end augmenting follicle number and restoring estrogen secreted by GCs to some extent. Similarly, UFM1 silencing was found to enhance ER stress but not oxidative stress-induced GC apoptosis during goat follicular development and atresia, suggesting a conserved role of UFMylation in the prevention of cell death related to the ER in ovaries (97).

## 4 Conclusions

Generally, the UFM1 modification cascade and its biological significance have not been well established and characterized although a number of studies laid the groundwork for the basic mechanisms underlying conjugation enzymes (5, 98, 99). In view of the expression patterns of UFMylation components in diverse tissues in mammals, recent work started to draw attention to an emerging role of UFM1 in endocrine glands as researchers continue to witness contributions of this ubiquitin-like modification to the sophisticated and fine-tuned activities of the ER network and the secretory pathway. Theoretically an endocrine mechanism delineates a body communication system acting on a distant target tissue through feedback loops of hormones released by internal glands, whose regulations heavily rely on a robust ER function of the secretory cells. In respect to this, we overviewed the latest advances in the maintenance of ER homeostasis by crosstalk from the UFM1 machinery based on a brief introduction of the ER-enriched UFMylation. In an attempt to strengthen the understanding of relevant cell physiology and human diseases, we focused on the interrelationship between UFM1 modification and ER stress-induced apoptosis in the context of endocrine glands pancreas and female ovaries. Nonetheless, whether UFMylation system is involved in other specialized endocrine organs, including hypothalamus, pituitary gland, pineal gland, thyroid gland, parathyroid gland, thymus gland, adrenal gland and testis, remains to be explored. Last but not the least, the properties of the few candidate UFMylation substrates reported to date, with those well-characterized in **Table 1**, do not readily explain the phenotypes associated with loss-of-function of the UFM1 pathway (31, 32, 100, 101). Studies in this direction may be invaluable for opening alternative avenues in promising therapeutic opportunities for endocrine disorders.

## Author contributions

All authors listed have made a substantial, direct and intellectual contribution to the work, and approved it for publication.
